# Seleno-detergent MAD phasing of leukotriene C_4_ synthase in complex with dodecyl-β-d-selenomaltoside

**DOI:** 10.1107/S1744309111042345

**Published:** 2011-11-29

**Authors:** Hiromichi Saino, Hideo Ago, Yoko Ukita, Masashi Miyano

**Affiliations:** a Structural Biophysics Laboratory, RIKEN SPring-8 Center, Harima Institute, 1-1-1 Kouto, Sayo, Hyogo 679-5148, Japan; b Department of Life Science, University of Hyogo, 3-2-1 Kouto, Kamighori, Akoh, Hyogo 678-1297, Japan; c Structural Biology Laboratory, Department of Chemistry and Biological Science, College of Science and Engineering, Aoyama Gakuin University, Fuchinobe 5-10-1, Chuo-ku, Sagamihara, Kanagawa 252-5258, Japan

**Keywords:** membrane proteins, MAD phasing, seleno-detergents, leukotriene C_4_ synthase

## Abstract

Dodecyl-β-d-selenomaltoside (SeDDM) is a seleno-detergent with a β-glycosidic seleno-ether in place of the ether moiety in dodecyl-β-d-maltoside. Seleno-detergents are candidates for heavy-atom agents in experimental phasing of membrane proteins in protein crystallography. Crystals of a nuclear membrane-embedded enzyme, leukotriene C_4_ synthase (LTC_4_S), in complex with SeDDM were prepared and a multiwavelength anomalous diffraction (MAD) experiment was performed. The SeDDM in the LTC_4_S crystal exhibited sufficient anomalous diffraction for determination of the structure using MAD phasing.

## Abstract 2

Dodecyl-β-d-selenomaltoside in a leukotriene C_4_ synthase crystal exhibited sufficient anomalous diffraction for multiwavelength anomalous diffraction phasing.

## 1. Introduction   

The discovery of heterologous expression has driven a rapid increase in the structure determination of membrane proteins. The number of membrane-protein structures identified using heterologous expression since 2007 is twice that identified from natural sources (Bill *et al.*, 2011 ▶). Recombinant techniques allow crystals of novel membrane proteins that cannot be obtained from natural sources to be prepared, which enables data collection for experimental phasing. This situation is similar to that of water-soluble proteins in the late 1980s. The dramatic increase in the structure determination of water-soluble proteins was achieved by MAD phasing methods using energy-tunable synchrotron-radiation X-rays and selenomethionine (SeMet) labelling of recombinant proteins to give heavy-atom derivatives, in conjunction with sophisticated computer hardware and software (Joachimiak, 2009 ▶)

SeMet is widely applicable for the preparation of heavy-atom derivatives owing to the diverse array of hosts for SeMet protein expression, which include *Escherichia coli*, yeast, insect cells and mammalian cells (Walden, 2010 ▶). Eukaryotic hosts favourably pro­duce functionally folded membrane proteins from higher organisms; however, the production of sufficient quantities of SeMet-labelled membrane proteins for crystallography remains a challenge owing to the time-consuming and expensive large-scale culture required, as well as the toxicity of SeMet. SeMet phasing needs a Met residue(s) for substitution, not including the N-terminal Met residue in the protein; therefore, it may be necessary to mutate certain residue(s) to methionine in the well ordered region (Ago *et al.*, 2007 ▶). There are still difficulties associated with the recombinant expression and SeMet labelling of membrane proteins. Derivatizations of membrane-protein crystals are achieved by extensive screening of heavy-atom agents.

Heavy-atom soaking and cocrystallization remain important methods for phasing membrane proteins that have novel folds (Morth *et al.*, 2006 ▶). To derivatize proteins, heavy metals or halogens have been used to prepare isomorphous derivative(s) of the covalent/noncovalent bonds at certain hydrophilic residues *via* soaking or cocrystallization methods. However, derivatized crystals frequently diffract less well than native crystals and the incorporation of heavy atom(s) occasionally causes non-isomorphism with the native crystal. These problems are particularly serious for labile membrane-protein crystals because native crystals of membrane proteins often diffract poorly.

**Figure 1:**
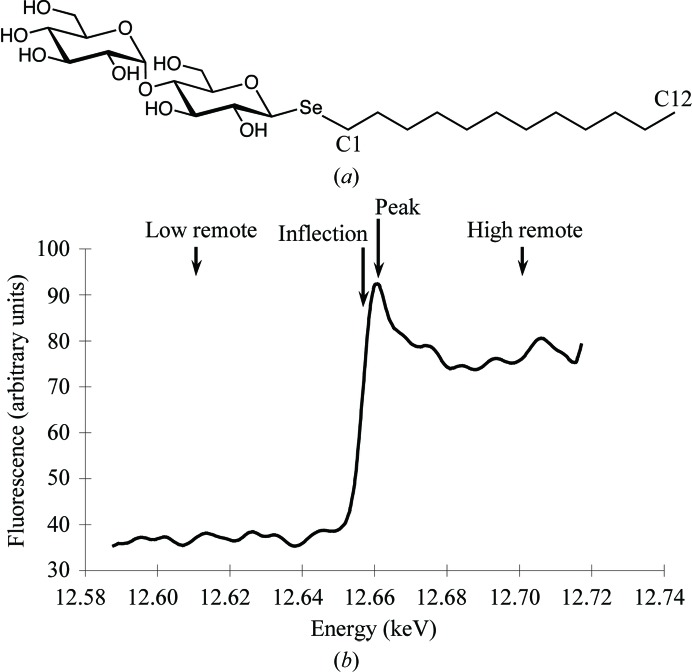
(*a*) The chemical structure of dodecyl-β-d-selenomaltoside (SeDDM). (*b*) Fluorescence scan of the Se *K* edge of the LTC_4_S crystal complex indicating the data-collection energies.

Dodecyl-β-d-selenomaltoside (SeDDM; Fig. 1 ▶ *a*) is seleno­substituted dodecyl-β-d-maltoside (DDM), which is a standard detergent for membrane-protein crystals. The substitution of oxygen by selenium maintains similar chemical properties, including geo­metry, because selenium and oxygen are in the same group of the periodic table (group 16). Therefore, this seleno-detergent may be a suitable heavy-atom derivative for membrane proteins, since DDM has been used for solubilization and crystallization (Sonoda *et al.*, 2011 ▶).

Detergent molecules are occasionally ordered in crystals; for example, 60 membrane-protein structures have been reported in complex with alkyl-β-d-maltosides or alkyl-β-d-glucosides (Appendix *A* [App appa]). This represents one fifth of the 299 unique membrane proteins among a total of 847 Protein Data Bank (PDB) entries (http://blanco.biomol.uci.edu/mpstruc/). This includes 12 membrane proteins complexed with DDM, 11 proteins complexed with nonyl-β-d-glucoside, 29 proteins complexed with octyl-β-d-glucoside and eight proteins complexed with other alkyl-β-d-maltosides (Appendix *A* [App appa]). These structures suggest that seleno-detergents are potentially applicable for experimental phasing of membrane proteins. Leukotriene C_4_ synthase (LTC_4_S) should be a good candidate to validate SeDDM phasing since we have previously solved the crystal structure of this membrane protein *via* X-ray crystallography with DDM (Ago *et al.*, 2007 ▶; Saino *et al.*, 2011 ▶). There are several defined DDM molecules per 17 kDa monomer of LTC_4_S and the biologically functional unit of LTC_4_S is a homotrimer.

In addition to its use in experimental phasing, the anomalous signal of the Se atom can act as a reference to more accurately define the position of SeDDM. The reference should be useful especially when the detergent molecule binds to functionally important sites of the membrane protein such as the lipid-binding site. In the crystal structure of LTC_4_S the DDM molecule occupies the active-site cleft at the interface of adjacent monomers (Ago *et al.*, 2007 ▶; Saino *et al.*, 2011 ▶). LTC_4_S catalyzes the conjugation of glutathione (GSH) and leukotriene A_4_ (LTA_4_), which is an unsaturated fatty acid that is involved in eicosanoid biosynthesis. The DDM binding site is the putative binding site of the substrate LTA_4_, since the alkyl chain of DDM resides in the hydrophobic cleft connected to the bound GSH site. Based on this DDM-binding mode, the LTA_4_-binding model was proposed using the DDM molecule as a substrate mimic (Ago *et al.*, 2007 ▶; Martinez Molina *et al.*, 2007 ▶). However, the position of the alkyl tail and glycosidic O atom of the DDM molecule remained ambiguous in the previous structure because the electron densities of oxygen and carbon could not be accurately discriminated even in the high-resolution structure (Saino *et al.*, 2011 ▶).

In this study, we formed complex crystals of SeDDM with LTC_4_S to show the applicability of SeDDM as a heavy-atom agent for MAD phasing. We successfully executed structural determination *via* MAD phasing of SeDDM. The anomalous electronic densities of the Se atoms provided a more defined structure of the SeDDM molecules that included the precise orientation of the alkyl chain in the proposed LTA_4_-binding cleft of LTC_4_S.

## 2. Materials and methods   

### 2.1. Purification and crystallization   

Human LTC_4_S was overexpressed by *Schizosaccharomyces pombe* with a His_6_ tag at the C-terminus and was purified using DDM as described previously (Ago *et al.*, 2007 ▶; Saino *et al.*, 2011 ▶). In brief, LTC_4_S was solubilized using a DDM/deoxycholic acid mixture and was purified using *S*-hexylglutathione affinity resin, Ni–agarose resin and size-exclusion chromatography. A PD-10 desalting column was equilibrated with 25 ml 0.04%(*w*/*v*) SeDDM (Affymetrix), 20 m*M* MES–NaOH pH 6.5, 5 m*M* GSH; 2.5 ml purified protein solution was then applied and eluted with 3.5 ml buffer for detergent exchange. The SeDDM-exchanged protein was concentrated to 6 mg ml^−1^ and stored at 193 K. Crystals of LTC_4_S with SeDDM were grown at 293 K using the sitting-drop vapour-diffusion method with equal amounts of protein solution and reservoir solution (0.1 *M* MES–NaOH pH 6.5, 1.6 *M* ammonium sulfate, 0.4 *M* MgCl_2_). The crystals were transferred into a harvesting solution that did not contain SeDDM (0.1 *M* MES–NaOH pH 6.5, 2.4 *M* ammonium sulfate, 50 m*M* GSH). The crystals were then dipped into a cryosolution supplemented with 15%(*v*/*v*) ethylene glycol and cooled in liquid nitrogen.

### 2.2. Data collection, processing and phasing   

MAD data were collected at wavelengths of 0.97909 Å (12.663 keV, peak), 0.97938 Å (12.659 keV, inflection), 0.97600 Å (12.703 keV, high-energy remote) and 0.98300 Å (12.613 keV, low-energy remote), as estimated from a fluorescence scan of the Se *K* edge, at 100 K using the BL26B2 beamline at the SPring-8 facility (Fig. 1 ▶ *b*). A total of 720 images were collected from one crystal, with 180 images for each wavelength and an oscillation of 1°. The data-collection statistics are given in Table 1 ▶. The images were processed using *MOSFLM* (Leslie, 1992 ▶; Winn *et al.*, 2011 ▶) and scaled using *SCALA* from *CCP*4 (Winn *et al.*, 2011 ▶). *SOLVE* in *PHENIX* (Adams *et al.*, 2010 ▶; Terwilliger, 2000 ▶) was used to determine the selenium substructure and calculate the initial phase; the resultant electron density was improved using *RESOLVE* in *PHENIX* (Adams *et al.*, 2010 ▶; Terwilliger, 2000 ▶). Structural refinement was carried out using *REFMAC*5 (Murshudov *et al.*, 2011 ▶; Winn *et al.*, 2011 ▶); *CNS* (Brünger *et al.*, 1998 ▶) was used for simulated annealing and *Coot* (Emsley *et al.*, 2010 ▶) was used for model building. The mean phase error of the initial phase angles against the model phases of the refined structure was calculated using *CPHASEMATCH* (Winn *et al.*, 2011 ▶).

## 3. Results and discussion   

### 3.1. Selenium MAD phasing   

**Figure 2:**
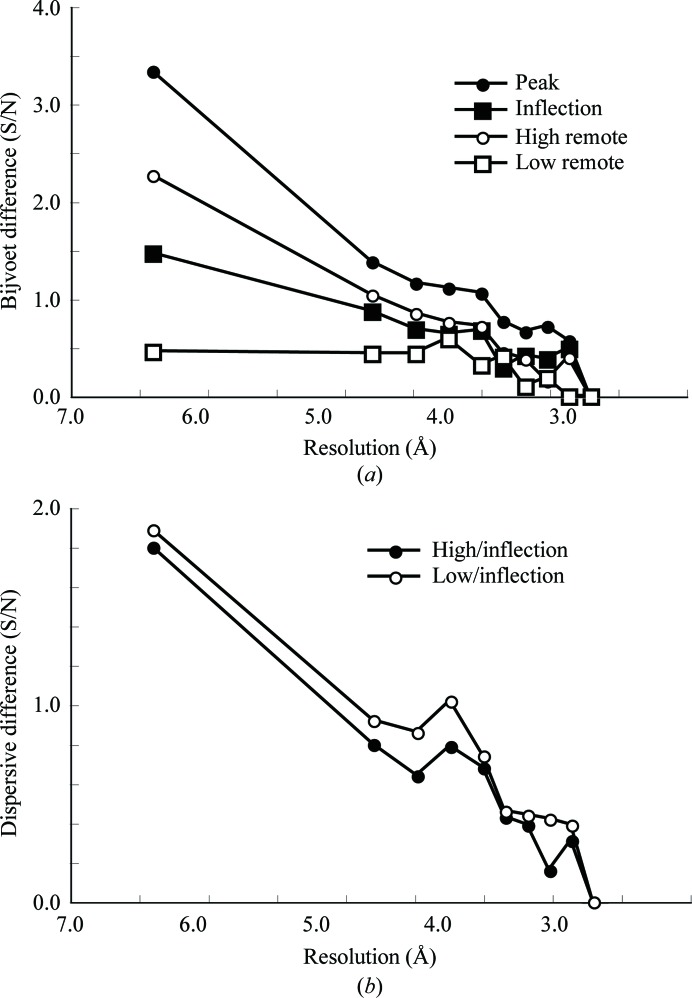
(*a*) Bijvoet difference signal-to-noise ratio (S/N) for the four MAD data sets. S/N ratios were calculated using *SOLVE* in *PHENIX* (Adams *et al.*, 2010 ▶; Terwilliger, 2000 ▶). (*b*) Dispersive difference (S/N) of the MAD data sets.

**Figure 3:**
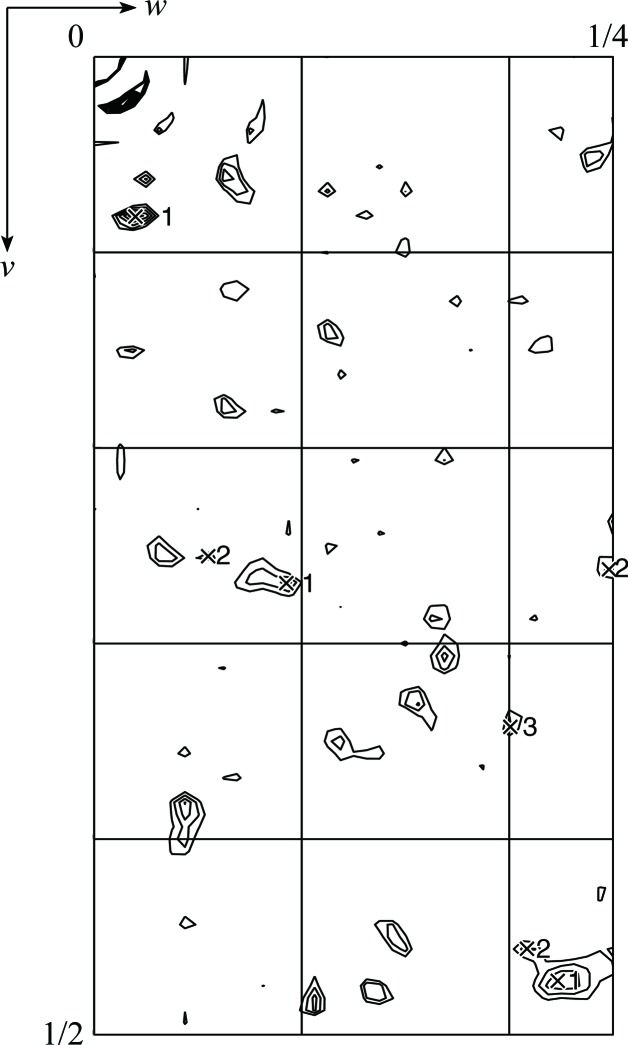
Anomalous difference Patterson map calculated from the peak data set at a resolution of 3.2 Å. The asymmetric area of the Harker section (*u* = 0) is drawn at contour levels from 1.5σ to 10σ in 0.5σ steps. The numbered crosses indicate symmetry-related self-vectors of the three selenium sites assigned using self-vectors calculated from the solution given by *SOLVE* in *PHENIX* (Adams *et al.*, 2010 ▶; Terwilliger, 2000 ▶).

The cocrystallized SeDDM in the LTC_4_S crystal gave a significant anomalous signal (Fig. 2 ▶). The signal-to-noise ratio was over 1.0 up to 4.0 Å resolution for the peak data set (Adams *et al.*, 2010 ▶; Terwilliger, 2000 ▶). The inflection and high-energy remote data also exhibited higher signal-to-noise ratios than the low-energy remote data set up to 4.0 Å resolution, which suggested that the anomalous contribution of the Se atoms in the diffraction data was statistically significant. The dispersive signals estimated with high-remote/inflection and low-remote/inflection data were comparable and the peak, inflection and high-energy remote data sets were used for substructure determination of the Se atoms. An anomalous difference Patterson map (Fig. 3 ▶) showed significant peaks corresponding to the selenium sites, as described below.

**Figure 4:**
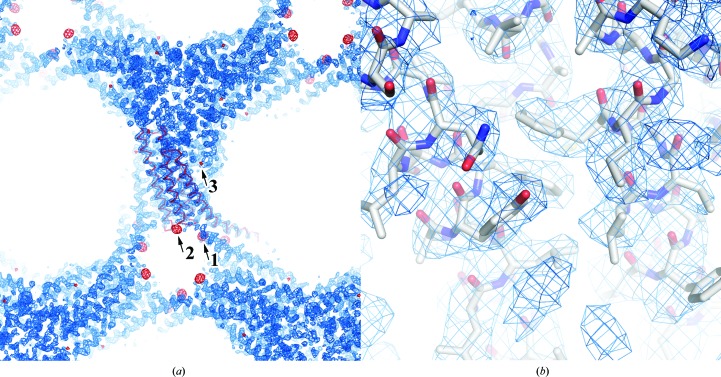
The electron-density map calculated from the experimental phases after density modification. (*a*) The blue mesh is the electron-density map contoured at 1.5σ. The red mesh is the anomalous difference Fourier map calculated from the experimental phases and the anomalous differences of the peak data set contoured at 5σ. The red ribbon shows the C^α^ trace of an asymmetric unit. (*b*) Magnified view of the electron-density map with the corresponding structure of the refined LTC_4_S model.

There were three selenium sites: two were found using *SOLVE* in *PHENIX* (Adams *et al.*, 2010 ▶; Terwilliger, 2000 ▶), while the other was manually picked from the anomalous difference Fourier map (Fig. 4 ▶). The first two sites had high occupancy in absolute scaling: 0.64 (*B* factor of 85.0 Å^2^) at site 1 and 0.54 (*B* factor of 86.9 Å^2^) at site 2, as determined by heavy-atom searching. The initial phases were calculated using these two heavy-atom positions and this was followed by phase improvement to 3.2 Å resolution. The corrected overall figure of merit was 0.75 and the solvent content was 68%. An anomalous difference Fourier map was calculated using the experimental phases of the two sites and the given corresponding peaks of 25σ and 15σ, respectively. Besides these two peaks, an additional peak corresponding to a minor site (6.3σ) was found. With the additional site incorporated, the occupancy and *B*-factor values refined to 0.41 and 63.0 Å^2^, respectively, for site 1, 0.28 and 61.1 Å^2^ for site 2 and 0.07 and 35.0 Å^2^ for site 3. The refined values of *f*′ and *f*′′ were −8.04 and 4.22, respectively, for the peak data, −9.16 and 2.25 for the inflection data and −5.53 and 3.81 for the high-remote data. In the anomalous difference Patterson peaks, self-cross-vector peaks were present with their symmetry-related positions; site 1 was the highest peak (Fig. 3 ▶). The phases were recalculated to include the additional site and applied to *RESOLVE* in *PHENIX* (Adams *et al.*, 2010 ▶; Terwilliger, 2000 ▶). The mean phase errors of the final model were 46.4° and 43.8° for the two-site and the three-site calculations, respectively (Adams *et al.*, 2010 ▶; Terwilliger, 2000 ▶). The latter phase angles are closer to those of the refined phases; this indicates that site 3 contributes substantially to phasing even though its occupancy is not high. The resultant election-density map was sufficient to unambiguously build an atomic model (Fig. 4 ▶).

*De novo* model building was carried out manually to avoid model bias from the previous DDM-complex structure of LTC_4_S (Ago *et al.*, 2007 ▶; Saino *et al.*, 2011 ▶). The structure of LTC_4_S in complex with SeDDM was refined using the low-remote data set to *R* = 0.203 and *R*_free_ = 0.210 at 3.2 Å resolution using *REFMAC*5 (Murshudov *et al.*, 2011 ▶; Winn *et al.*, 2011 ▶; Table 1 ▶). The root-mean-square deviation between the refined structure and the previous high-resolution structure (PDB entry 3pcv; Saino *et al.*, 2011 ▶) was 0.62 Å for all atoms.

### 3.2. SeDDM binding sites   

**Figure 5:**
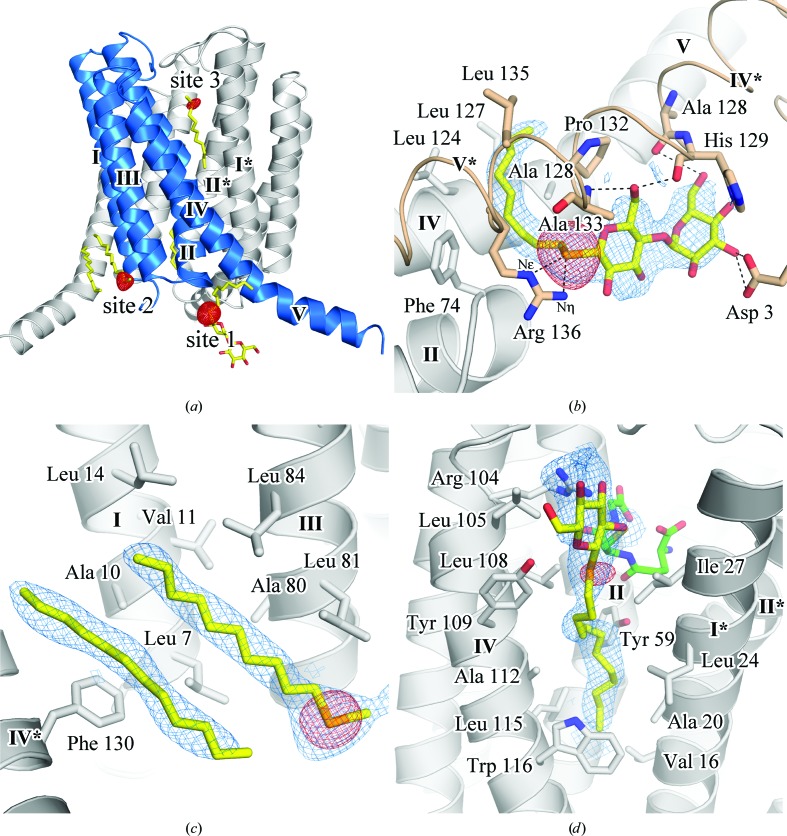
(*a*) SeDDM molecules in an LTC_4_S trimer with anomalous difference Fourier map. The red mesh is the anomalous difference Fourier map calculated from the refined phases and the anomalous differences of the peak data set contoured at 5σ of an asymmetric unit. SeDDM is represented by a stick model with yellow C atoms. The blue cartoon model indicates a monomer in the LTC_4_S trimer and the roman numerals show the sequential order of the helices. Magnified views of the SeDDM binding sites are shown in (*b*), (*c*) and (*d*), with the electron densities of the SeDDM molecules represented by a blue mesh contoured at 1.0σ. (*b*) SeDDM and its surrounding residues in site 1. The dashed lines indicate hydrogen bonds between SeDDM and amino-acid residues. The wheat-coloured models labelled IV* and V* indicate helices IV and V of a twofold-symmetry-related molecule in the crystal packing. (*c*) SeDDM of site 2. The neighbouring alkyl chain and its election density are also displayed. (*d*) SeDDM in site 3. The bound glutathione is represented by green C atoms.

There were three SeDDM binding sites with selenium peaks in the anomalous difference Fourier map (Fig. 5 ▶ *a*). Site 1 was between helices IV and V and helices IV* and V* in a twofold symmetry-related molecule. Site 2 was along the transmembrane helices I and III, and site 3 was in the active site of LTC_4_S. The alkyl chain of SeDDM in each site was bound in the same mode as that of the corresponding DDM in the previous structures (PDB entries 3pcv, 3leo, 2pno and 2uuh; Saino *et al.*, 2011 ▶, Rinaldo-Matthis *et al.*, 2010 ▶; Ago *et al.*, 2007 ▶; Martinez Molina *et al.*, 2007 ▶). These results indicate that SeDDM molecules bound competitively with DDM molecules.

At site 1 the Se atom was located close to the guanidino group of Arg136 at distances of 3.3 and 3.5 Å to N^η^ and N^∊^, respectively (Fig. 5 ▶ *b*). The maltoside of SeDDM was surrounded by polar groups, *i.e.* Asp3 of helix I*, the carbonyl O atom of Ala128, the N^δ^ and carbonyl O atoms of His129 of helix IV* and the backbone amide of Ala133 of helix V* (Fig. 5 ▶ *b*). Electron density for the alkyl chains was observed for the C1–C9 C atoms; the alkyl chains were close to (*i.e.* within 4 Å of) hydrophobic residues, including Phe74, Leu124, Leu127, Ala128, Pro132 and Leu135.

The strong peak (25σ) in the anomalous difference Fourier map indicates that the selenium is stably located at this site. In addition, the electron densities corresponding to the maltose moiety were clearer with SeDDM than with DDM (PDB entries 2pno, 2uui, 2uuh, 3leo and 3pcv; Ago *et al.*, 2007 ▶; Martinez Molina *et al.*, 2007 ▶; Rinaldo-Matthis *et al.*, 2010 ▶; Saino *et al.*, 2011 ▶). These results suggest that polar interactions with selenium and maltoside, such as hydrogen bonding, provide tighter binding of SeDDM than DDM.

The SeDDM in site 2 (Fig. 5 ▶ *c*) was surrounded by hydrophobic residues, *i.e.* Leu7, Ala10, Val11 and Leu14 in helix I and Ala80, Leu81 and Leu84 in helix III. These residues were within 4.2 Å of the alkyl chain of SeDDM. Electron densities for the Se atom and alkyl chains were clearly observed, whereas that of the maltose moiety was not definitive. According to the orientation of the alkyl chain and the Se atom, the maltose moiety beyond the seleno-ether must extend into the solvent. Electron density corresponding to the putative alkyl chain together with the SeDDM molecule of site 2 was present, but no anomalous peak was observed (Fig. 5 ▶ *c*).

Site 3 is the putative LTA_4_-binding site in the active site of LTC_4_S. The anomalous peak of the Se atom overlapped with the end of the long electron density of the C1–C12 alkyl chain, which indicates the position of the seleno-ether (Fig. 5 ▶ *d*). The alkyl chain was inserted into the valley between helices composed of hydrophobic residues, including Leu105, Leu108, Tyr109, Ala112, Leu115 and Trp116 of helix IV, Tyr59 of helix II and Val16, Ala20, Leu24 and Ile27 of helix I* in the adjacent monomer. The electron density of maltoside was located next to the thiol group of the GSH.

The electron density of the alkyl chain of SeDDM was consistent with the previous DDM structure and the LTA_4_-binding model (Ago *et al.*, 2007 ▶). The LTA_4_-binding model was constructed based around the alkyl chain of DDM. The aliphatic chain of LTA_4_ was embedded along the alkyl chain (C12) of DDM at the bottom of the cavity covered by the indole ring of Trp116 (Ago *et al.*, 2007 ▶; Martinez Molina *et al.*, 2007 ▶; Rinaldo-Matthis *et al.*, 2010 ▶; Saino *et al.*, 2011 ▶). This binding model implies that SeDDM and DDM affect the activity of LTC_4_S: experimentally, both SeDDM and DDM showed inhibitory activity against LTC_4_S catalysis in a preliminary enzyme assay (data not shown).

## 4. Conclusion   

The SeDDM in the LTC_4_S crystal provided sufficient anomalous signal for selenium MAD phasing. The alkyl chains of the SeDDM molecules were surrounded by hydrophobic residues in all three sites, indicating that hydrophobic interactions are involved in the binding of SeDDM. This work suggests that SeDDM is applicable for phase determination. DDM molecules were found in the complex structures of 11 membrane proteins (Appendix *A* [App appa]); their alkyl chains also form hydrophobic interactions in their binding sites. The molecular weight per detergent ratio (kDa per detergent molecule; Appendix *A* [App appa]) shows that several membrane proteins bind a larger number of detergent molecules than LTC_4_S.

The alkyl chains of SeDDM formed hydrophobic interactions similar to those of DDM in the previously reported LTC_4_S structures. The SeDDM molecules bound three sites in competition with DDM molecules; therefore, it is possible that the DDM-binding sites were not fully substituted by SeDDM. Detergent exchange by a more thorough method, such as washing with SeDDM on an affinity column, may be necessary if the anomalous signal is insufficient.

In addition to experimental phasing, the anomalous scattering of the Se atom in the seleno-detergent allows the binding mode of these detergent molecules to be defined more accurately than with common detergents. The positions of the Se atoms and linked alkyl chains were confirmed by using the anomalous peaks as positional references. The position of the SeDDM alkyl chain in the putative LTA_4_-binding site was clearly defined. This result supports the previous LTA_4_-binding model based on the binding of DDM in the active site (Ago *et al.*, 2007 ▶).

## Supplementary Material

PDB reference: LTC_4_S with SeDDM, 3b29

Structure factors: contains datablock(s) r3b29sf. DOI: 10.1107/S1744309111042345/wd5163sup1.hkl

## Figures and Tables

**Table 1:** Data-collection, phasing and refinement statistics Values in parentheses are for the highest shell.

	Low remote	High remote	Peak	Inflection
Unit-cell parameters ()	*a* = *b* = *c* = 168.6	*a* = *b* = *c* = 168.8	*a* = *b* = *c* = 168.5	*a* = *b* = *c* = 168.7
Space group	*F*23			
Wavelength ()	0.98300	0.97600	0.97909	0.97938
Resolution ()	26.73.20 (3.373.20)	26.73.20 (3.373.20)	26.63.20 (3.373.20)	26.73.20 (3.373.20)
Total No. of images	180	180	180	180
*R*_merge_	0.13 (0.37)	0.13 (0.40)	0.13 (0.40)	0.13 (0.37)
Completeness (%)	89.9 (69.3)	90.0 (68.9)	90.0 (69.5)	90.0 (68.8)
Multiplicity	21.9 (21.1)	21.9 (22.2)	21.9 (22.1)	21.9 (22.2)
*I*/(*I*)	5.6 (2.1)	5.3 (1.9)	5.4 (1.9)	5.3 (2.1)
Phasing				
No. of Se sites			3	
FOM			0.24	
Refined *f*/*f*		5.53/3.81	8.04/4.22	9.61/2.55
Density modification				
Solvent content (%)			68	
FOM			0.75	
Refinement				
Resolution range ()	25.43.20			
*R*/*R*_free_	0.203/0.210			
*B* factor (^2^)				
Protein	30.5			
Ligands	31.1			
Detergents	62.3			
Se site 1	68.5			
Se site 2	94.5			
Se site 3	59.0			
R.m.s. deviations				
Bond lengths ()	0.002			
Bond angles ()	0.511			
Chiral volumes (^3^)	0.032			
Ramachandran plot				
Favoured (%)	95.2			
Allowed (%)	4.8			
Disallowed (%)	0			

**Table 2:** Membrane-protein structures with modelled alkyl--D-glucoside and alkyl--D-maltoside in the PDB kDa/detergent indicates the molecular weight of protein per detergent molecule in the asymmetric unit.

Detergent (PDB residue code)	Unique†	Name	PDB code	Structure weight (kDa)	No. of detergent molecules	kDa/detergent (kDa)
Octyl--D-glucoside (BOG)	29‡	Ram prostaglandin H_2_ synthase-1 (COX-1; *Ovis aries*)	1pth	133	2	66
			1cqe	133	5	27
			1eqh	133	3	44
			1ht5	127	3	42
			1ht8	127	3	42
			1eqg	133	3	44
			1q4g	127	8	16
			2ayl	127	7	18
			3n8w	127	3	42
			3n8x	127	2	64
			3n8y	127	4	32
			3n8z	127	5	25
			3n8v	127	5	25
		Glycerol-3-phosphate dehydrogenase (GlpD, native; *Escherichia coli*)	2r4j	114	6	19
			2r45	114	6	19
			2r46	114	5	23
			2r4e	114	6	19
			2qcu	114	6	19
		Dihydroorotate dehydrogenase in complex with atovaquone (*Rattus rattus*)	1uum	81	2	40
		Omp32 anion-selective porin (*Delftia acidovorans*)	2fgq	35	1	35
		OmpF matrix porin in complex with colicin peptide OBS1 (*E. coli*)	3o0e	232	1	232
		OmpG monomeric porin in open state (*E. coli*)	2iww	66	12	5
		VceC outer membrane protein (*Vibrio cholerae*)	1yc9	48	1	48
		OmpT outer membrane protease (*E. coli*)	1i78	67	4	17
		OmpLA (PldA) outer membrane phospholipase A monomer	1qd5	32	5	6
			1ild	31	5	6
			1im0	31	4	8
			1ilz	31	5	6
		Cytolysin pore-forming toxin protomer (*V. cholerae*)	1xez	80	1	80
		Sensory rhodopsin II (SRII; *Natronomonas pharaonis*)	1jgj	23	1	23
			1h2s	30	1	30
		Rhodopsin in meta II state (bovine rod outer segment)	3pqr	40	2	20
			3pxo	39	2	20
			3dqb	40	3	13
		Rhodopsin (squid)	2z73	100	2	50
		M2 proton channel (influenza A)	3bkd	22	6	4
		SLAC1 anion channel, TehA homologue (wild type; *Haemophilus influenzae* A)	3m73	35	4	9
			3m74	35	4	9
			3m75	35	4	9
			3m76	35	4	9
			3m71	35	4	9
		AQP4 aquaporin water channel (human)	3gd8	24	1	24
		AqpM aquaporin water channel (*Methanothermobacter marburgensis*)	2evu	25	2	13
		AqpZ aquaporin water channel (*E. coli*)	2o9g	24	2	12
			3nka	48	4	12
			3nkc	48	3	16
			3nk5	48	4	12
		GlpF glycerol facilitator channel (*E. coli*)	1fx8	30	3	10
			1ldi	30	2	15
			1lda	30	2	15
			1ldf	30	2	15
		PfAQP aquaglyceroporin (*Plasmodium falciparum*)	3c02	28	1	28
		Aqy1 yeast aquaporin (pH 3.5; *Pichia pastoris*)	2w1p	30	4	7
			2w2e	30	6	5
		FocA formate transporter without formate (*V. cholerae*)	3klz	153	14	11
			3kly	153	16	10
		AmtB ammonia channel (mutant; *E. coli*)	1u7g	40	1	40
			2ns1	56	8	7
		Rh protein, possible ammonia or CO_2_ channel (*Nitrosomonas europaea*)	3b9z	41	2	20
			3b9y	41	2	20
			3b9w	43	1	43
		Human Rh C glycoprotein ammonia transporter (*Homo sapiens*)	3hd6	54	1	54
		LeuT_*Aa*_ leucine transporter (*Aquifex aeolicus*)	2a65	58	5	12
			2q6h	58	5	12
			2qb4	58	5	12
			2qei	58	5	12
			3f4j	58	4	15
			3f48	58	6	10
			3f3e	58	7	8
			3f3d	58	5	12
			3f4i	58	5	12
			3f3c	58	5	12
			3f3a	58	7	8
			2qju	57	4	14
			3gjc	115	8	14
			3gjd	58	6	10
			3mpq	57	3	19
			3mpn	57	6	9
		Oestrone sulfatase (human placenta)	1p49	63	2	32
		Cytochrome *bc*_1_ (*Gallus gallus*)	2bcc	229	1	229
			1bcc	229	1	229
		Light-harvesting complex (*Rhodopseudomonas acidophila*)	1nkz	31	6	5
Nonyl--D-glucoside (BNG)	11‡	Archaerhodopsin-2 (aR-2; *Halorubrum* sp. aus-2)	1vgo	55	12	5
		Rhodopsin (bovine rod outer segment; *Bos taurus*)	1hzx	78	7	11
			1l9h	78	7	11
		Kir3.1 prokaryotic Kir chimera (*Mus musculus*/*Burkholderia xenovorans*)	2qks	72	1	72
		AQP0 aquaporin water channel (bovine lens)	1ymg	28	2	14
		AQP1 aquaporin red blood cell water channel (*B. taurus*)	1j4n	29	3	10
		GlpG rhomboid-family intramembrane protease (*E. coli*)	2ic8	21	12	2
			3b44	20	17	1
			3b45	20	17	1
			2o7l	20	1	20
			2xow	20	16	1
			2xov	20	19	1
			2xtu	20	18	1
		FucP fucose transporter in outward-facing conformation (*E. coli*)	3o7p	48	1	48
			3o7q	48	1	48
		UraA uracil/H^+^ symporter (*E. coli*)	3qe7	45	1	45
		AdiC arginine:agmatine antiporter (*E. coli*)	3l1l	47	1	47
			3rlb	42	11	4
		Cytochrome *ba*_3_ (*Thermus thermophilus*)	1ehk	85	3	28
		Light-harvesting complex LHC-II, spinach photosystem II (*Spinacia oleracea*)	1rwt	250	10	25
Nonyl--D-maltoside (ZDM)	1	ChbC EIIC phosphorylation-coupled saccharide transporter (*Bacillus cereus*)	3qnq	192	4	48
Decyl--D-maltoside (DMU)	3	CorA Mg^2+^ transporter (*Thermotoga maritima*)	2bbh	32	4	8
		Cytochrome *c* oxidase, *aa*_3_ (bovine heart mitochondria)	1v55	410	2	205
			1v54	410	2	205
		Cytochrome *c* oxidase, two-subunit catalytic core (*Rhodobacter sphaeroides*)	2gsm	185	10	19
Dodecyl--D-maltoside (LMT)	12‡	Sulfide:quinone oxidoreductase in complex with decylubiquinone (*A.aeolicus*)	3hyv	285	6	48
			3hyx	285	6	48
			3hyw	285	6	48
		Prokaryotic pentameric ligand-gated ion channel (GLIC; *Gloeobacter violaceus*)	3eam	181	6	30
			3p4w	182	6	30
			3p50	182	6	30
		GluCl anion-selective receptor (Fabivermectin complex; *Caenorhabditis elegans*)	3rif	431	3	144
			3ri5	431	3	144
			3ria	431	3	144
			3rhw	431	3	144
		GlpG rhomboid-family intramembrane protease (*E. coli*)	2irv	41	1	41
		MexB bacterial multidrug efflux transporter (*Pseudomonas aeruginosa*)	2v50	682	8	85
		Leukotriene C_4_ synthase in complex with glutathione (human)	2pno	209	57	4
			2uui	17	2	9
			2uuh	17	1	17
		NrfH cytochrome *c* quinol dehydrogenase (*Desulfovibrio vulgaris*)	2j7a	795	6	132
		Fumarate reductase complex (*Wolinella succinogenes*)	1qlb	260	2	130
			2bs2	261	2	131
		Cytochrome *c* oxidase, *aa*_3_ (*Paracoccus denitrificans*)	3ehb	123	12	10
			3hb3	123	14	9
		Photosystem II (*Thermosynechococcus elongatus*)	1s5l	305	2	152
			2axt	304	6	51
			3bz2	306	7	44
			3bz1	306	7	44
		Photosystem II (*T. vulcanus*)	3arc	295	12	25
Undecyl--D-maltoside (UMQ)	4	Rotor of V-type Na^+^-ATPase (*Enterococcus hirae*)	2bl2	160	22	7
		Cytochrome *bc*_1_ (*Saccharomyces cerevisiae*)	1kb9	244	1	244
			1p84	244	1	244
			3cxh	520	2	260
			3cx5	520	2	260
		Cytochrome *b*_6_ *f* complex (*Mastigocladus laminosus*)	2e75	108	4	27
			2e76	108	4	27
			2e74	108	4	27
		Cytochrome *b*_6_ *f* complex (*Nostoc* sp. PCC 7120)	2zt9	106	3	35

† The unique number of membrane proteins counts the same proteins from different species differently.

‡ There are two membrane-protein structures in complex with two kinds of alkyl--D-glycosides each, *i.e.* BOG/BNG and BNG/LMT.

## Appendix A 60 membrane-protein structures with modelled alkyl-­β-­d-­glucoside or alkyl-β-d-maltoside in the PDB

There are 299 unique membrane-protein structures in 847 PDB entries for membrane proteins (http://blanco.biomol.uci.edu/mpstruc/). The total molecular weight of the protein and number of detergents in the asymmetric unit were investigated for 141 of the detergent-containing structures (Table 2 ▶).
